# Supplementary material for: Diauxie and co-utilization of carbon sources can coexist during bacterial growth in nutritionally complex environments
Source: Nat Commun. 2020 Jun 19;11:3135. doi: 10.1038/s41467-020-16872-8 (PMC7305145; doi:10.1038/s41467-020-16872-8)
Supplement: Supplementary file 1 — Supplementary Information [file 41467_2020_16872_MOESM1_ESM.pdf]

Supplementary Information

Diauxie and co-utilization of carbon sources can coexist during bacterial growth in nutritionally complex environments

Elena Perrin[1†], Veronica Ghini [2†], Michele Giovannini[1], Francesca Di Patti[3,4], Barbara Cardazzo[5], Lisa Carraro[5], Camilla Fagorzi[1], Paola Turano [6], Renato Fani[1], Marco Fondi[1*]

[1] Dep. of Biology, University of Florence, Italy
[2] Consorzio Interuniversitario Risonanze Magnetiche di Metallo Proteine (CIRMMP), Italy
[3] Dep. of Physics and Astronomy, University of Florence, Italy
[4] CSDC, University of Florence, Italy
[5] Dep. of Comparative Biomedicine and Food Science, University of Padova, Italy
[6] Center of Magnetic Resonance (CERM), University of Florence, Italy

* Corresponding author: marco.fondi@unifi.it
† Equal contributors

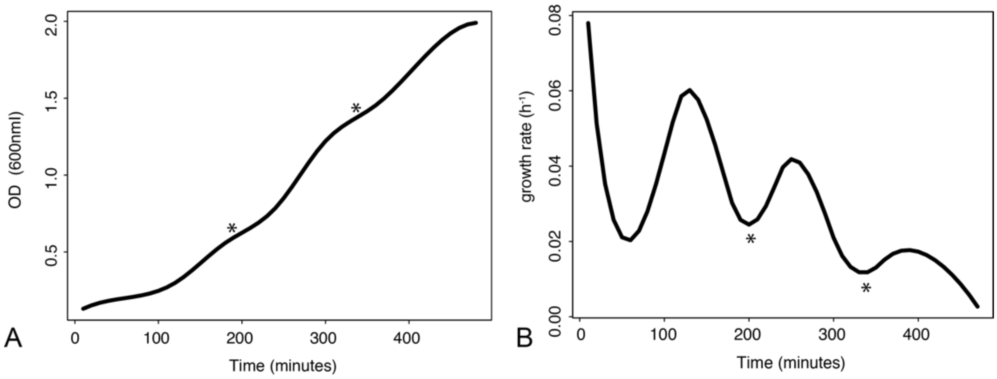

Supplementary Figure 1. Curve fitting on interpolated data: OD (A) and growth rates (B) estimation based on interpolated growth data.

Supplementary Table 1. Main features of the sequencing run obtained in this work.

Time point	***Replicate***	***N. of reads***	***NCBI accession code***	***File name***
T2	1	45589444	SAMN12207305	1_S14_R1_001.fastq.gz
T2	2	28516257	SAMN12207306	2_S1_R1_001.fastq.gz
T2	3	20484151	SAMN12207307	3_S2_R1_001.fastq.gz
T2	4	26905601	SAMN12207308	4_S3_R1_001.fastq.gz
T4	1	31632871	SAMN12207309	5_S4_R1_001.fastq.gz
T4	2	46766051	SAMN12207310	6_S15_R1_001.fastq.gz
T4	3	44460436	SAMN12207311	7_S16_R1_001.fastq.gz
T4	4	43330805	SAMN12207312	8_S5_R1_001.fastq.gz
T5	1	31713940	SAMN12207313	9_S6_R1_001.fastq.gz
T5	2	46981096	SAMN12207314	10_S17_R1_001.fastq.gz
T5	3	45117020	SAMN12207315	11_S18_R1_001.fastq.gz
T5	4	53527022	SAMN12207316	12_S19_R1_001.fastq.gz
T6	1	32100196	SAMN12207317	17_S10_R1_001.fastq.gz
T6	2	32739316	SAMN12207318	18_S11_R1_001.fastq.gz
T6	3	28202275	SAMN12207319	19_S12_R1_001.fastq.gz
T6	4	34848944	SAMN12207320	20_S13_R1_001.fastq.gz
T7	1	52496534	SAMN12207321	13_S20_R1_001.fastq.gz
T7	2	45537877	SAMN12207322	14_S7_R1_001.fastq.gz
T7	3	36699228	SAMN12207323	15_S8_R1_001.fastq.gz
T7	4	25020206	SAMN12207324	16_S9_R1_001.fastq.gz

Supplementary Note 1. Comparison with *E. coli* starvation regulators

Over-expressed TFs included a controller of outer membrane gene expression (OmpR), curli production activator (CsgD) and a putative siderophore transport system permease protein (YfhA). Among down-regulated genes we found the gene coding for stringent starvation protein response (*sspA*). In *E. coli* this protein is involved in resistance during

prolonged starvation (including amino acid starvation) as a positive regulator of many stress-related gene. Its expression is induced during stationary phase as well as upon starvation for carbon, amino acid, nitrogen and phosphate. Additionally, *sspA* is positively regulated by *relA* [1]. In our experiment, *relA* did not change its expression significantly. Together with *relA*, *spoT* is also responsible for the accumulation of cellular ppGpp (promoted by carbon source starvation but not by amino acid starvation). Even in this case, we did not notice a significant change in *spot* expression during our experiment. Besides the afore mentioned RpoS, up-regulated TFs included an OmpR member (PSHAa0628), putatively annotated as a response regulator consisting of a CheY-like receiver domain and a winged-helix DNA-binding domain. This sequence shares significant similarity (E-value 7e-41) with proteobacterial (i.e. *E. coli*) members of the two-component regulatory system CreC/CreB involved in catabolic regulation. PSHAa0622 encodes the response regulator GlrR, belonging to the GntR regulator family and up-regulating the transcription of the *glmY* sRNA when cells enter the stationary growth phase. Finally, the AraC transcriptional regulator PSHAa1588 is the fifth most up-regulated TF following T1-T3 transition, but no reliable functional annotation could be retrieved for this gene.
As for down-regulated TFs, *iscR* (PSHAa2672) is the one with the highest fold-change (-1.91). IscR is a transcriptional repressor of the *iscRSUA* operon, involved in assembly of Fe-S clusters. According to RegPrecise database, it is predicted to regulate the expression of 11 genes. PSHAa2995 (log2FC = -1.47), the forth most down-regulated gene putatively encodes a GlpR homolog, a transcriptional regulators of sugar metabolism. The third most down-regulated TF resulted to be PSHAa0390, encoding *pdhR*, the repressor of the pyruvate dehydrogenase complex. According to RegPrecise database, it is predicted to regulate the expression of 12 genes, all of them involved, at different stages in pyruvate metabolism. In *E. coli* the decreased pyruvate concentration means a decreased inhibition of PdhR [2]. While PdhR represses the transcription of its target genes, the pyruvate-bound state of the regulator is not able to bind DNA. PdhR controls (among the others) the transcription of the multi-enzyme complex of the pyruvate dehydrogenase complex [3]. Only one TF resulted to be differentially expressed (up-regulated) following the second lag phase, PSHAa1181, the sigma factor PSHAa0691.

Together with TFs, two-component regulatory systems (TCRSs) are a basic stimulus-response coupling mechanism to sense and react to changes in environmental conditions, e.g. nutrient concentration. For this reason, we identified differentially expressed TCRSs in the two selected contrasts. Overall, we found 21 TCRSs-related genes that were differentially expressed in T1 vs. T2. PSHAa0620 was the TCRS displaying the higher $\log_2$FC (1.9) following T1 to T2 transition, encoding a signal transduction histidine kinase belonging to the BaeS family, putatively involved in cell envelope stress response. PSHAa0134 (1.19 $\log_2$FC following T1-T2 transition) belongs to the VieA TCRS family and embeds both a REC and an EAL domain. The first is typical of two-component signal transduction systems enabling bacteria to sense, respond, and adapt to a wide range of environments, stressors, and growth conditions, whereas the second might be involved in regulating cell surface adhesiveness in bacteria [4].
Among the other over-expressed TCRS, we identified PSHAa2620, encoding a PleD-like response regulator, a two-component response regulator embedding two REC domains and a diguanylate cyclase (GGDEF) domain. Members of this family are the best-characterized regulator of c-di-GMP levels and motility in *C. crescentus* and have similarly been shown to be involved in regulating surface motility in other bacterial species [5, 6, 7, 8]. PSHAa0628 (the fourth most expressed TCRS) encodes a proteobacterial dedicated sortase system response regulator (*pdsR*). This family of DNA-binding response regulator proteins are usually associated with an adjacent histidine kinase to form a two-component [9]system;

PSHAa0628 makes no exception as it is flanked by PSHAa0629, a signal transduction histidine kinase (COG0642). The other up-regulated TCRS representatives included a nitrate/nitrite response regulator NarL (PSHAa2948), an osmolarity sensing histidine kinase (PSHAa2849), a CheY-like regulator (PSHAa1501) and the copper resistance/phosphate regulon response regulator CusR (PSHAb0012).
The most down-regulated TCRS member identified was PSHAb0361 an already characterized C4-dicarboxylate sensor kinase, probably involved in regulating the expression of a C4-dicarboxylate transporter system comprising PSHAb0363 and PSHAb0364 [9].

Supplementary Table 2. Differentially expressed global metabolic regulators in *Ph*TAC125. Adjusted p-values were calculated using Deseq2 default approach, i.e. correcting for multiple testing using the Benjamini and Hochberg method.

Locus tag	Product name	Function	Log_{10} FC	Adj. P-value
PSHAa2850	OmpR	regulator participates in controlling the expression of major outer membrane genes	1.01	8.37e-22
PSHAa2120	CsgD	activator of the csgBA and csgDEFG operons necessary for curli production	1.34	1.03e-11
PSHAa0622	YfhA	putative two component transcriptional regulator	1.82	3.68e-66
PSHAa0346	Fis	regulator of stable RNA (rRNA and tRNA) synthesis	-2.17	2.86e-45
PSHAa0390	LldR_GlcC_PdhR	Lactate regulator A transcription factor that controls expression of genes involved in transport and catabolism of L-lactate	-1.67	9.88e-72
PSHAb0078	CspA	cold shock protein; transcriptional activator of hns	-1.34	1.21e-11
PSHAa2568	HdfR	negatively regulates the expression of the flagellar master operon, flhDC A direct activator of the flhDC operon and represses transcription of hdfR	-1.15	5.77e-23
PSHAa2527	SspA	stringent starvation protein	-0.98	2.12e-31
PSHAa1641	Fur	regulator of ferric uptake	-0.97	2.39e-11

**PSHAa0838**	FadR	regulator of fatty acid metabolism	-0.70	3.00e-12

Supplementary Table 3. Global transcriptional regulators in *E. coli*, their ortholog in *Ph*TAC125 and whether they were differentially expressed or not in our experiment.

E. coli **regulator**	***E. coli*** **locus tag**	***Ph*****TAC125 closest homolog**	**DEG in *Ph*TAC125 (T1 vs. T2/T3 vs. T4)**
mlc	b1594	PSHAb0149	No/No
rpoS (sigma 38)	b2741	PSHAa0691	Yes/Yes
FecI (sigma 19)		-	
rpoE (sigma 24)	b2573	PSHAa0726	No/No
fliA (sigma 38)	b1922	PSHAa0809	No/No
rpoH (sigma 32)	b3461	PSHAa0357	No/No
rpoN (sigma 54)	b3202	PSHAa2551	No/No
rpoD (sigma 70)	b3067	PSHAa0349	Yes/No

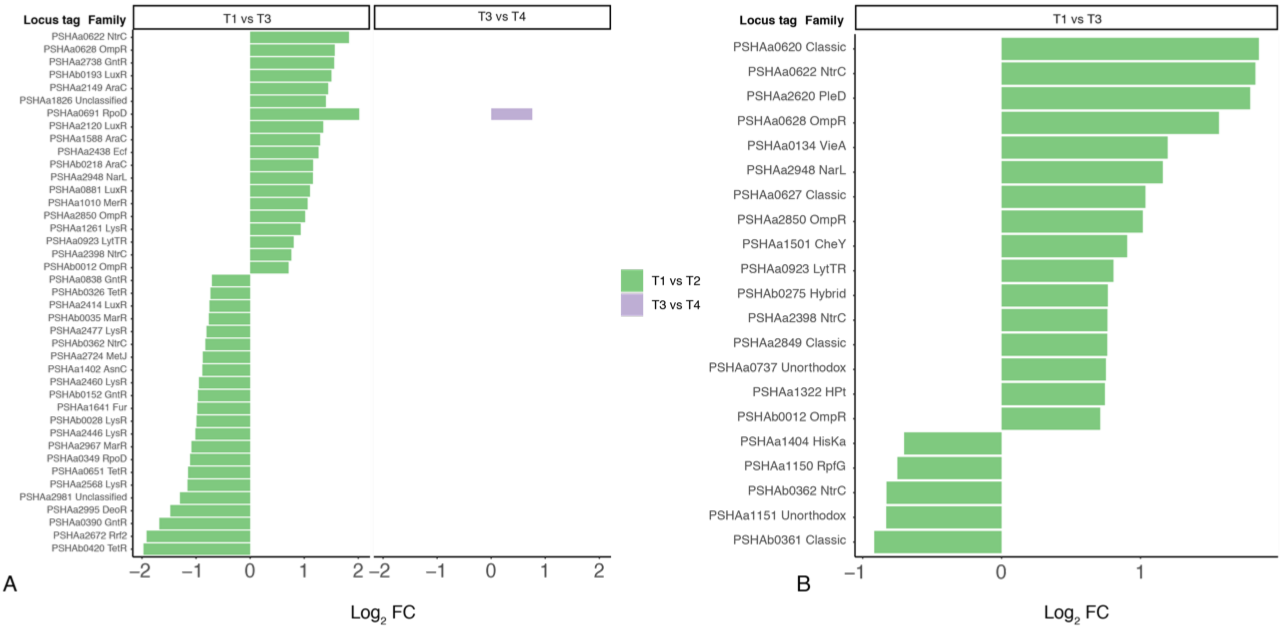

Supplementary Figure 2. Analysis of differentially expressed TFs and TCRSs. Differentially expressed transcription factors (A) and two-component regulation systems (B) in PhTAC125 following T1-T3 and T3-T5 transitions.

Supplementary Note 2. Main players of amino acid metabolism in a complex medium

The set of amino acids that are typically consumed in the first ours of PhTAC125 growth comprises Ser, Thr, Asp, Asn and Glu. Afterwards, PhTAC125 metabolism switches to the consumption of a second set of amino acids that includes Lys, Leu, Ala, Gly, Phe, Tyr, Ile and Val. In the experimental set up described by Wilmes et al. (2010) [10], this switch occurs after 4 hours. Four additional hours are then required to consume this second set of amino acids, and to finally start degrading the amino acid His. No information is currently available on the faith of Met, Cys, Trp, Pro, Gln and Arg during growth of PhTAC125 in a complex medium.

Indeed, Ser, Thr, Asn, Asp and Glu were shown to be the first amino acids to be consumed in an amino acid rich medium; consistently, we found the down-regulation of *purA*, *murI*, and *sdaA* involved in the degradation of Asp, Glu and Ser, respectively. Interestingly, 4 genes out of 7 of the down-regulated and 4 out of 6 of the up-regulated between T1 and T3 are involved in the first utilization step of the same amino acids, i.e. Asp, Glu, Met and Ser (Figure 2E). Considering the genes analysed here, Asp seems to be converted to andenylosuccinate at T1, then this gene is turned off and the degradation redirected towards the production of Arg. Similarly, the gene converting Ser to pyruvate (*sdaA*, L-serine ammonia-lyase) is predicted to be significantly down-regulated following T1 to T3 transition, whereas the genes involved in the conversion of Ser to Gly (*glyA*) shows a significant increase in the same contrast. Glutamate racemase *(murI)*, responsible for the conversion of L-Glu to D-Glu appears to be turned off between T1 and T3, whereas the expression of the gene responsible for the conversion of Glu to 2-oxo-glutarate (*gdhA*) shows a significant increase. Finally, *mdeA* and *metK* are significantly up- and dow-regulated, respectively, when considering the T1 vs T3 time interval. The first is responsible for the conversion of Met to methanediol, allowing the entrance of this intermediate into sulfur metabolism, whereas the second encodes the conversion of Met to S-adenosyl-methionine (SAM).

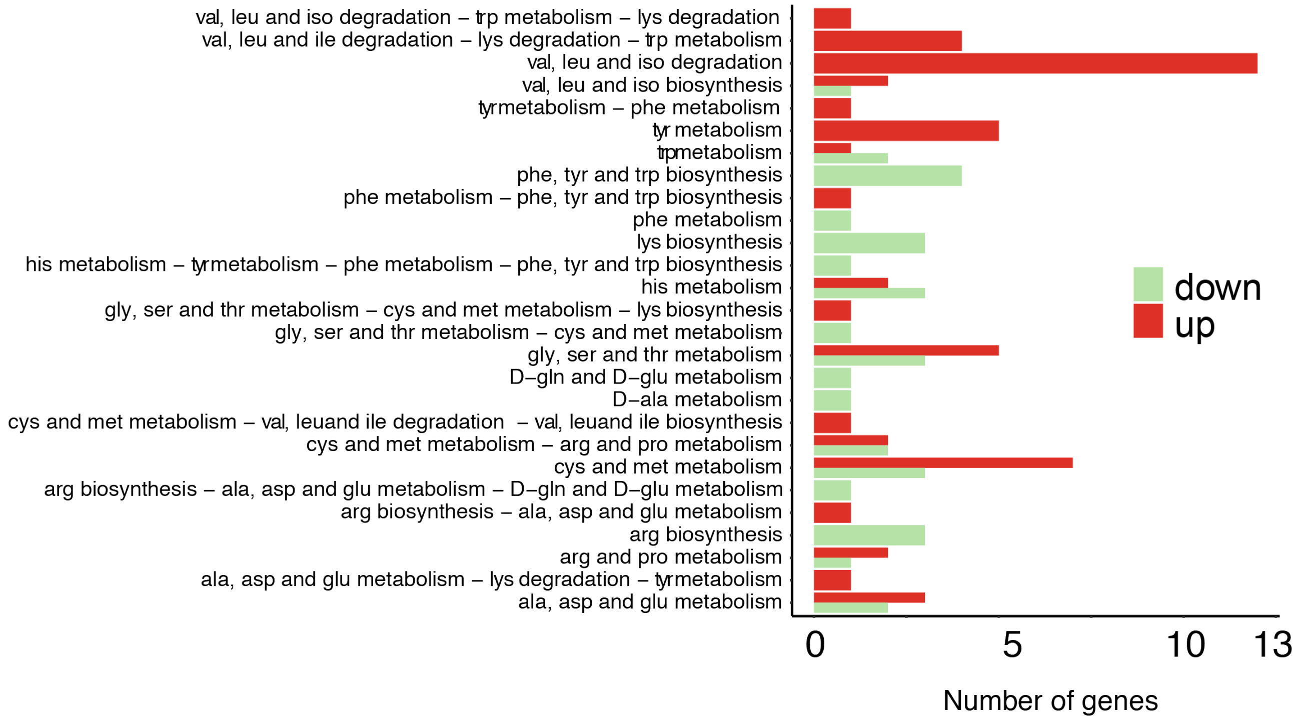

Supplementary Figure 3. Differentially expressed metabolic genes. Number of differentially expressed amino acid metabolic genes for each pathway

Supplementary Note 3. Amino acids metabolic pathways regulation.

The expression of all the genes involved in AA metabolism in PhTAC125 was evaluated (Supplementary Figure 3). Each gene was associated to a specific amino acid metabolic pathway according to the KEGG database and the correlation (Pearson correlation) among each gene belonging to the same pathway was evaluated (Supplementary Table 4). In most cases, we found a low level of (positive) correlation among the expression values of the genes belonging the same metabolic pathway. Most of the Fisher's z-transformed correlation coefficient range between 0.5 and 0.14. Genes involved in Tyr metabolism and branched chain amino acid degradation are the ones that display the highest correlation values, with 1.15 and 1.07. respectively. Conversely, Phe, Tyr and Trp biosynthetic genes were those with the lowest correlation (0.14).

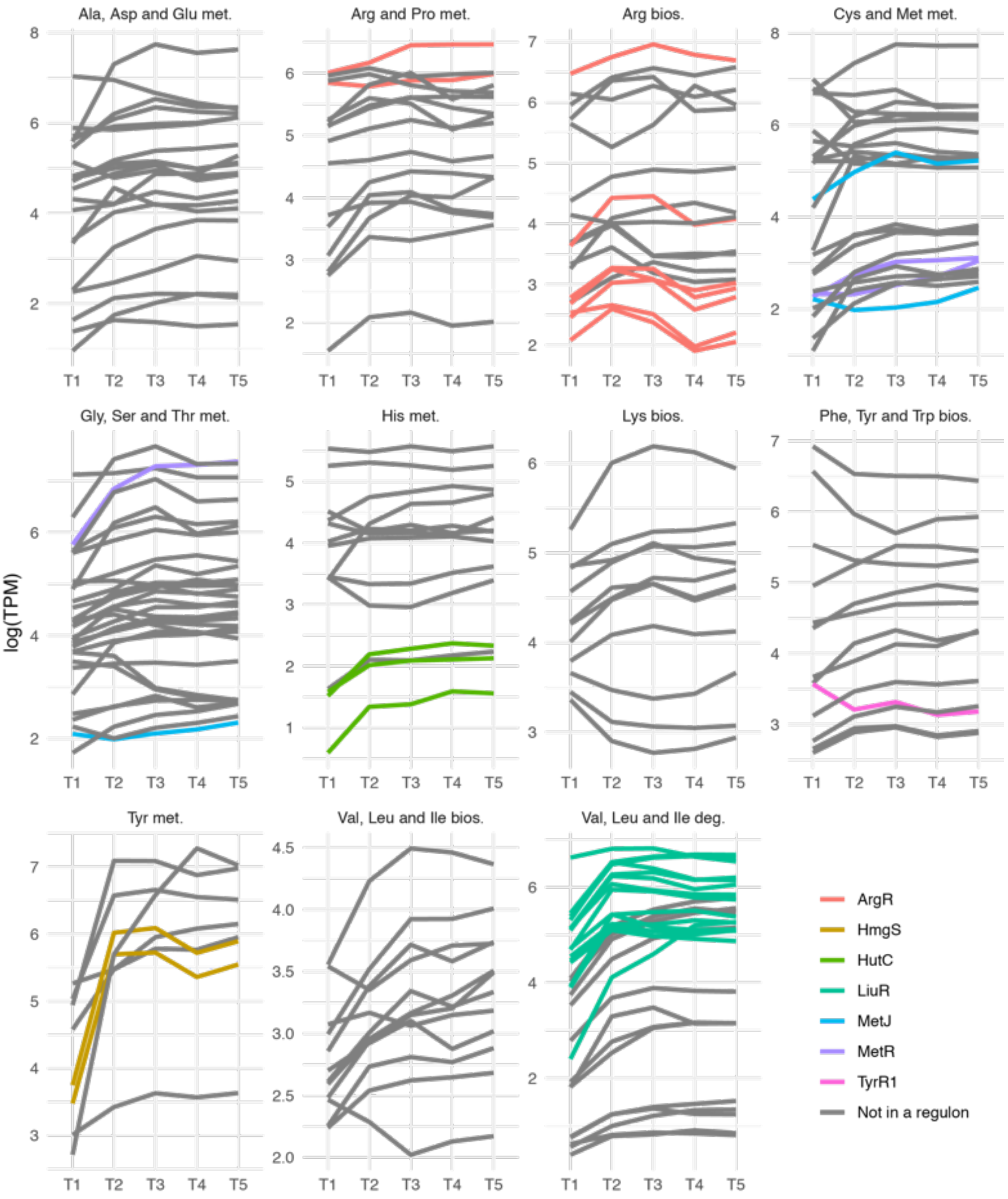

Supplementary Figure 4. Expression of amino acids metabolic genes. Gene expression pattern of all the genes involved in amino acid metabolism in *Ph*TAC125. Coloured lines represent genes assigned to a specific regulon (see legend) according to the RegPrecise database.

Supplementary Table 4. Fisher's Z transformation average of Pearson correlation coefficient among the genes belonging to the same amino acid metabolic pathway.

**Amino acid metabolic pathway**	**Fisher's z transformation of Pearson correlation coefficient**
Tyr met.	1,15
Val, Leu and Ile deg.	1,07
Val, Leu and Ile bios.	0,59
Arg and Pro met.	0,511
Ala, Asp and Glu met.	0,51
Gly, Ser and Thr met.	0,44
Cys and Met met.	0,29
Arg bios.	0,271
His met.	0,23
Lys bios.	0,23
Phe, Tyr and Trp bios.	0,14

Supplementary Table 5. Main information for all the *Ph*TAC125 regulons considered in this work.

Regulator	Effector	Pathway	Operons
ArgR	Arginine	Arginine biosynthesis; Arginine degradation	4
HmgS		Tyrosine degradation	2
HutC	cis-Urocanic acid	Histidine utilization	2
LiuR		Branched-chain amino acid degradation	9
MetJ	S-adenosylmethionine	Methionine metabolism; Methionine biosynthesis	9
MetR	Homocysteine	Methionine biosynthesis	4
TyrR1	Tyrosine; Phenylalanine	Aromatic amino acid metabolism	4

Supplementary Note 4. Constraint based metabolic modelling of PhTAC125 growth in a nutritionally rich environment and comparison with transcriptomics data.

In a previous work [11], we have simulated the growth of PhTAC125 in a nutritionally complex environment (peptone) and derived the overall metabolic reprogramming occurring during growth in a rich undefined medium. More in detail, we previously identified ten distinct phases in the growth of PhTAC125 on peptone medium, each of them corresponding to a

time step of one hour. For each of these phases we identified the specific uptake rates of the different compounds (amino acids) present in the growth medium and/or (possible) switches in the use of the available C-sources. An implicit assumption of this method is that fluxes are considered to be constant within these intervals.
Computed uptake rates were used as input for ten different FBA simulations (selecting biomass production as the objective function) to derive the most likely fluxes distribution in the PhTAC125 metabolic model in each time step. Since nutritional condition resembles the one used in the first experiment of this work and from which transcriptomics data was gathered we checked whether the observed gene expression profiles for the different AA regulons (Figure 3B) are consistent with the fluxes predicted using constraint-based metabolic modelling. Specifically, we monitored the changes in predicted metabolic fluxes during a multi-step FBA growth on a simulated peptone medium for those reactions whose genes were included in the AA regulons of PhTAC125. Additionally, we have also compared the observed gene expression profiles for the different AA regulons with the *ad hoc* implemented approach named nutritional-MOMA, an approach that uses the minimization of the metabolic adjustments required at each (metabolic) transition of the entire growth period analysed. Overall, we found a good agreement between gene co-expression and fluxes distribution for genes/reactions belonging to the same regulon both when using the previously implemented multi-step FBA (Pearson's product-moment correlation = 0.89, p-value = 0.019) and nutritional-MOMA approach (Pearson's product-moment correlation= 0.85, p-value = 0.030).

Supplementary Table 6. Fisher's Z transformation average of Pearson correlation coefficient among the genes belonging to the same regulon and time-resolved, predicted fluxes obtained during constraint-based metabolic modelling on a simulated undefined rich medium.

Regulon	*Process*	*Fisher's Z transformation of Pearson correlation coefficient*		
		Gene Expression	Nutritional MOMA	Multi-step FBA
HutC	His degradation	2,55	Inf	Inf
HmgS	Tyr degradation	1,92	-	-
MetR	Met biosynthesis	1,22	0,42	0,8
LiuR	Branched-chain amino acid degradation	0,97	0,34	0,56
ArgR	Arg biosynthesis/degradation	0,47	1,27	1,17
MetJ	Met metabolism, Met degradation	0,26	0,48	0,64
TyrR1	Aromatic amino acids metabolism	0,11	0,36	0,14

Supplementary Note 5. NMR-based metabolomic analysis of 19-AA medium.
In order to determine the kinetics of the 19 AA (0.2 mM each) usage during PhTAC125 growth, we analysed the growing media composition over time using ^{1}H NMR, Supplementary Figure 5.

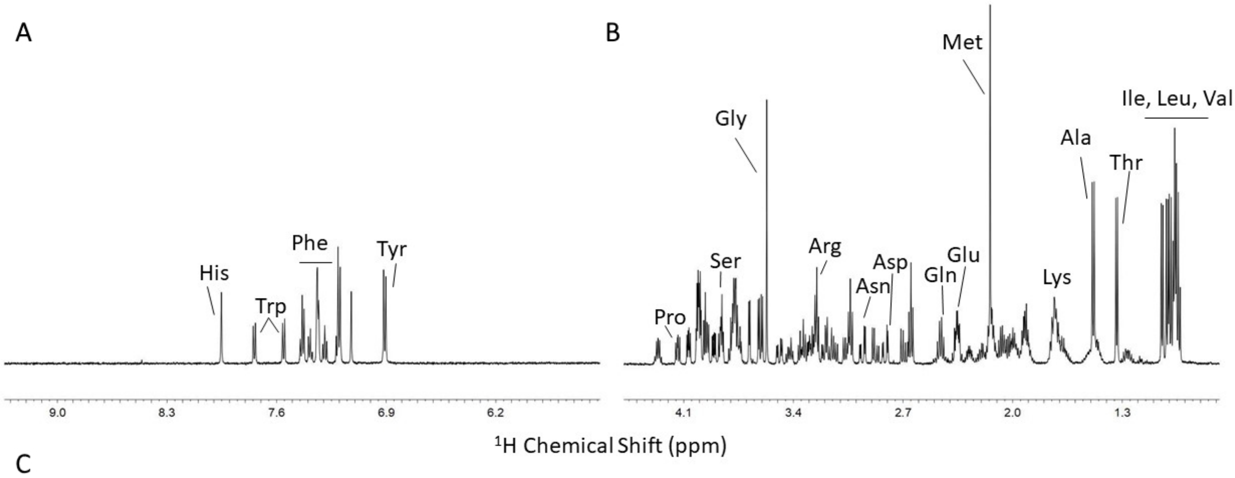

C

**Val**	0.98 (d)	1.03 (d)	2.26 (m)	3.60 (d)				
**Leu**	0.95 (t)	1.70 (m)	3.72 (m)					
**Ile**	0.93 (t)	1.00 (d)	1.25 (m)	1.46 (m)	1.97 (m)	3.66 (d)		
**Thr**	1.32 (d)	3.58 (d)	4.24 (m)					
**Met**	2.16 (m)	2.63 (t)	3.85 (dd)					
**Ala**	1.47 (d)	3.77 (q)						
**Gln**	2.12 (m)	2.45 (m)	3.77 (t)					
**Glu**	2.04 (m)	2.12 (m)	2.34 (m)	3.75 (dd)				
**Asp**	2.67 (m)	2.83 (m)	3.87 (dd)					
**Asn**	2.84 (m)	2.94 (m)	4.00 (dd)					
**Lys**	1.46 (m)	1.71 (m)	1.89 (m)	3.02 (t)	3.74 (t)			
**Arg**	1.68 (m)	1.90 (m)	3.23 (t)	3.76 (t)				
**Pro**	2.00 (m)	2.08 (m)	2.34 (m)	3.32 (dt)	3.41 (m)	4.12 (dd)		
**Gly**	3.54 (s)							
**Ser**	3.83 (dd)	3.96 (m)						
**Tyr**	3.02 (dd)	3.17 (dd)	3.92 (dd)	6.88 (m)	7.17 (m)			
**His**	3.16 (dd)	3.23 (dd)	3.98 (dd)	7.09 (d)	7.9 (d)			
**Trp**	3.29 (dd)	3.47 (dd)	4.05 (dd)	7.19 (m)	7.27 (m)	7.31 (s)	7.53 (d)	7.72 (d)
**Phe**	3.11 (m)	3.27 (m)	3.98 (m)	7.37 (m)				

Supplementary Figure 5. **Metabolomics data for the 19 AA experiment. A)** Upfield (1.00–4.50 ppm) and **B)** downfield (5.50–9.00 ppm) region of the ^{1}H NMR NOESY spectrum of 19 AA (0.2 mM) growing medium. **C)** ^{1}H NMR resonance assignment for the 19 amino acid' signals. s= singlet; d= doublet; t=triplet; dd= doublet of doublets; dt= doublet of triplets; m=multiplet.

Supplementary Note 6: Relationship between the order of consumption of amino acids and the growth of PhTAC125 when using single amino acids as single C source.

We investigated the correlation between amino acids consumption and the achievable biomass amount when the same carbon sources of the 19 AA experiment were used as single carbon and energy sources. For this reason, we used available growth phenotypes. More specifically, Supplementary Figure 6A refers to data obtained in another work (see

reference [29] in the main text) using Phenotype Microarray and reflected the achievable OD after 167 hours when PhTAC125 cells were grown using each single amino acid as the sole carbon and energy source. To obtain this figure, we retrieved the ODs for all the C1, C2, C3 and C4 amino acids separately and then built the box plot shown in Supplementary Figure 6A. Similarly, Supplementary Figure 6B reports growth rates of 4 amino acids from a previous work (see reference [23] in the main text) when these four amino acids were used as sole C and energy source in a fed-batch growth. Also, in this case, the order of consumption was derived from the metabolomic data obtained in the present work. However, for this analysis, to infer the order of consumption, we didn't rely on the cluster of each amino acid but we considered the time in which the concentration of each amino acid reached the value of 0. Supplementary Figure 6A shows that, on average, amino acids included in cluster 1 permit a higher cell density than those in cluster 2 and so on. Similarly, when focusing on single amino acids and the growth rate measured when using them as unique C source, higher growth rates are obtained with those amino acids that are degraded first in our 19 amino acids growth curve (Supplementary Figure 6B). These data suggest that, when faced with multiple alternatives, bacterial metabolism has evolved in order to start feeding on those that ensure then most efficient growth, leaving the others for the latter stages of the growth.

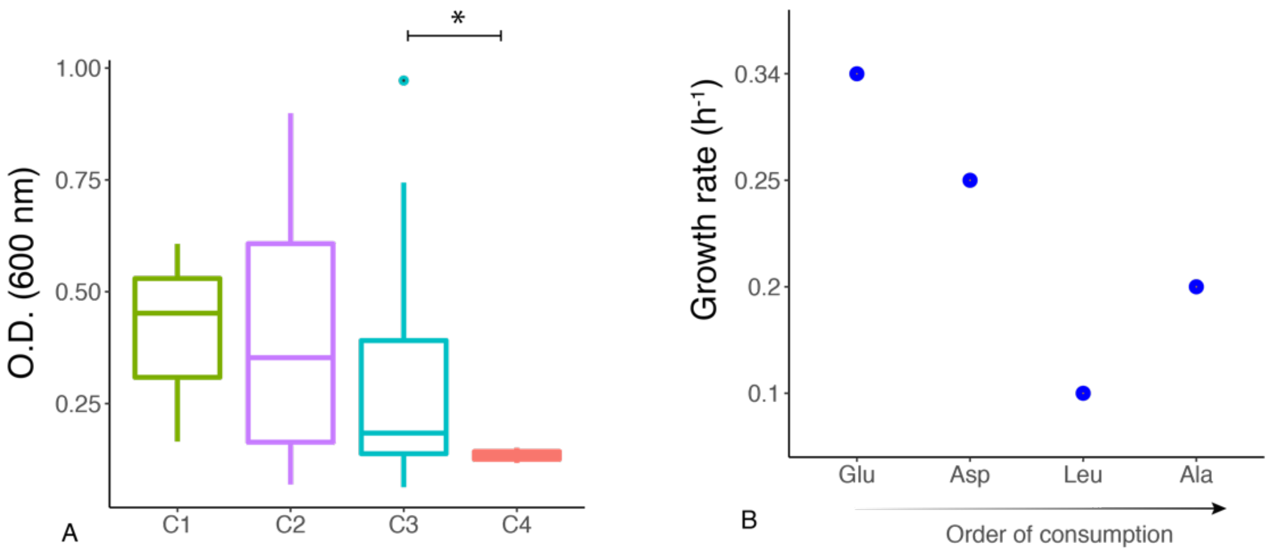

Supplementary Figure 6. Relationship between the order of amino acids uptake and growth features. A) Average obtained biomass when using each of the amino acids of each cluster as sole carbon source. Symbols give the median (horizontal line inside the box), central 50% range (box margins), range (vertical line) within inner fences (1.5 times box range from box margins) and outliers (dots). The asterisk denotes the statistically significant comparison, using "two sided" Pearson's product moment correlation coefficient **B)** Growth rates obtained when using the amino acids indicated on the x-axis as sole carbon source. Source data are provided as a Source Data file.

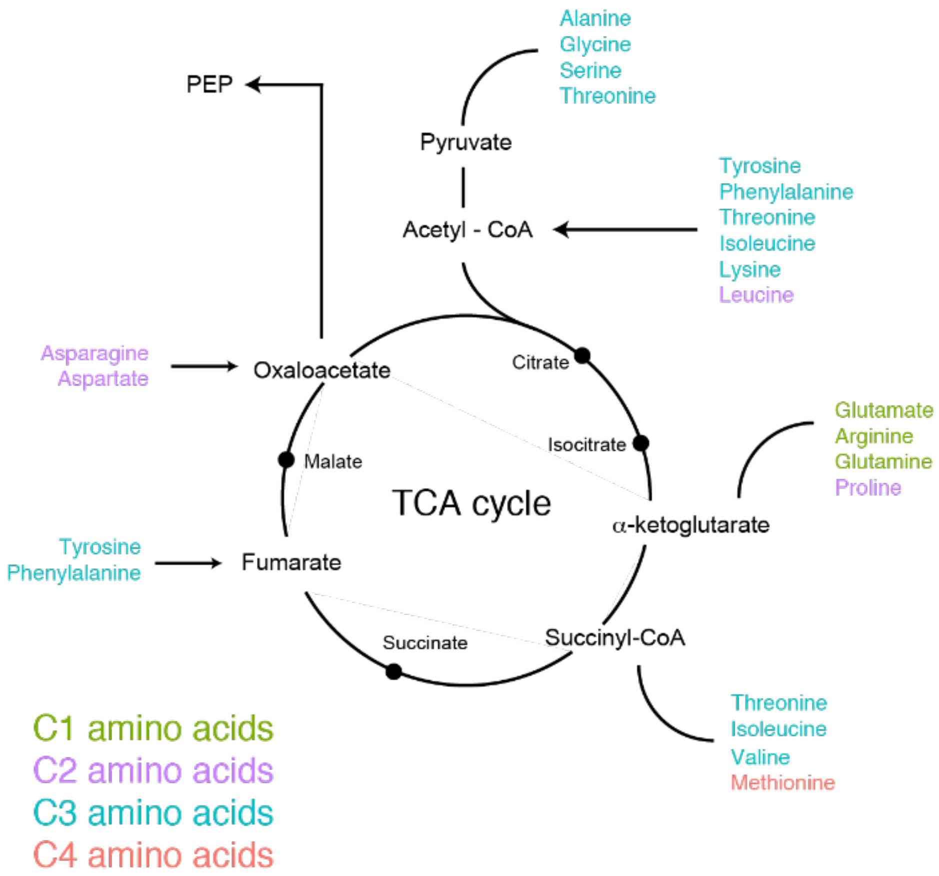

Supplementary Figure 7. Entry points of the 19 amino acids. Schematic representation of C1, C2, C3 and C4 amino acids entry points into PhTAC125 metabolism. Only those amino acids that are effectively metabolised by PhTAC125 during the 19 AA experiment are shown here.

Supplementary Note 7. Relationship between amino acids entry points into PhTAC125 metabolism and their order of consumption

We checked whether the order of amino acids uptake matched their entry point into PhTAC125 metabolism. Despite a few exceptions (that could be explained by, for instance, the expression of broad-range amino acids transporters), the order of consumption of amino acids mirrors the possible entry points into the TCA. Indeed, group 1 and 2 amino acids enter the TCA through the formation of alpha-ketoglutarate or oxaloacetate (Supplementary Figure 7). More specifically, all the Group 1 amino acids (plus Pro from group 2) enters the TCA though the formation of alpha-ketoglutarate whereas the Asp and Asn (Group 2) amino acids enter through the formation of oxaloacetate. With the exception of His (whose consumption is overall negligible according to our metabolomic data) no Cluster 3 or Cluster 4 amino acid enters the TCA through these two intermediates. Amino acids from these two latter clusters enter the TCA through the formation of succinyl-CoA, pyruvate, acetyl- or acetoacetyl-CoA.

Supplementary Note 8. Nutrients concentration does not influence the main clusters of assimilated amino acids

To test whether the sequential or co-consumption of substrates was dependent upon their own concentration, we increased the concentration of the amino acids in the 19-AA

experiment described in the main text and traced their concentration in time. More specifically, we performed two experiments in which the concentration of all the amino acids was 5- and 10-times higher than the original one (0.2 mM), respectively. The results of these experiments are reported in Supplementary Figure 8.

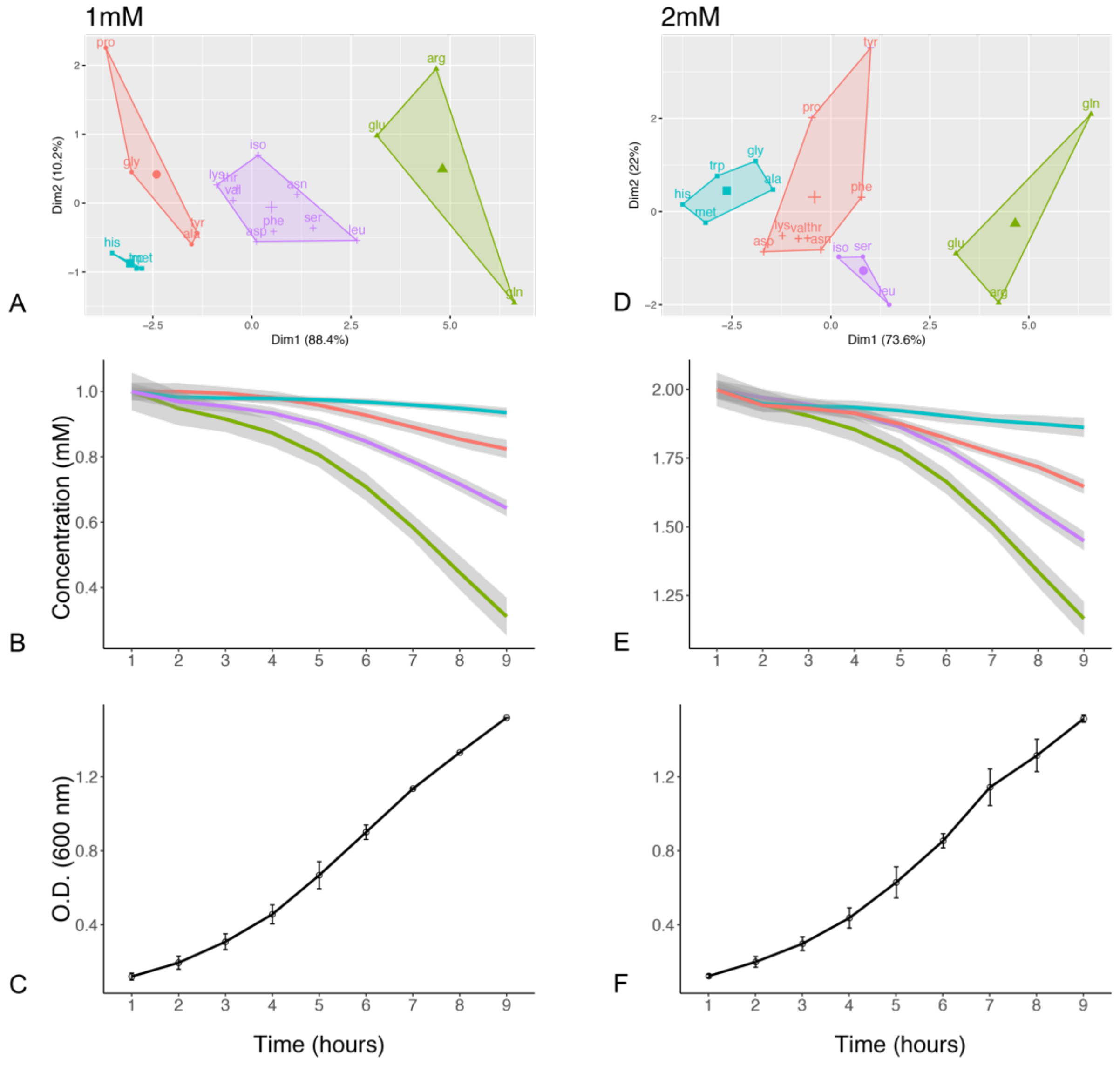

Supplementary Figure 8. Amino acids degradation patterns in the 19 AA experiment. A) Clustering of time-resolved concentration values for the 19 amino acids at a 1 mM concentration. **B)** Degradation dynamics for each of the 4 identified clusters of amino acids included in the defined AA 1mM initial concentration. Grey shaded area includes the 95% confidence of the linear regression (coloured) line over the concentrations of the amino acids belonging to the same group. **C)** Growth curve of the 1 mM 19 AA experiment. Error bars represent SD of 2 different cell cultures in 2 independent experiments. **D-F)** report the same measures for the same experiments with all amino acids at an initial concentration of 2 mM. Source data are provided as a Source Data file

Overall, these experiments confirmed the results obtained when growing PhTAC125 on the 19 AA medium at a concentration of 0.2 mM in that the amino acids belonging to the same

group are simultaneously metabolized by cells but the assimilation of different groups occurs with slightly different dynamics. Concerning the clustering of the amino acids into different groups according to their assimilation kinetics, we observed an overall scenario that resembled the one observed in the 0.2 mM experiment. Cluster 1 (C1) amino acids (Glu, Gln and Arg) are readily and quickly metabolized by PhTAC125, regardless of their initial concentration. Conversely, amino acids belonging to Cluster 4 (C4, Met, Trp and His) are not used by growing PhTAC125 cells, at least in the analysed time frame. This suggests that these latter amino acids are probably assimilated only upon exhaustion of the other 16, as observed in the 0.2 mM 19-AA experiment. In-between these two major clusters, the remaining 13 amino acids are consumed with slightly different rates in all the performed experiments and, as a result, producing different groups during the clustering (Figure 4 and Supplementary Figure 8).

To analyse whether the “canonical” diauxic shift observed during the 19-AA experiment was influenced by the concentration of the involved amino acids (i.e. the C4 amino acids, Met, Trp and His), we also performed an experiment in which the concentration of this final group of compounds largely exceeded the one of the other amino acids. Specifically, we provided these three amino acids at higher concentration (2 mM) and checked whether this influenced their order of uptake in the same 19 AA medium used before. This new experimental condition didn’t influence the major split between early-metabolized amino acids (Cluster 1 to 3 of the original experiment) and the late-metabolized ones (Cluster 4). These data are reported in Supplementary Figure 9.

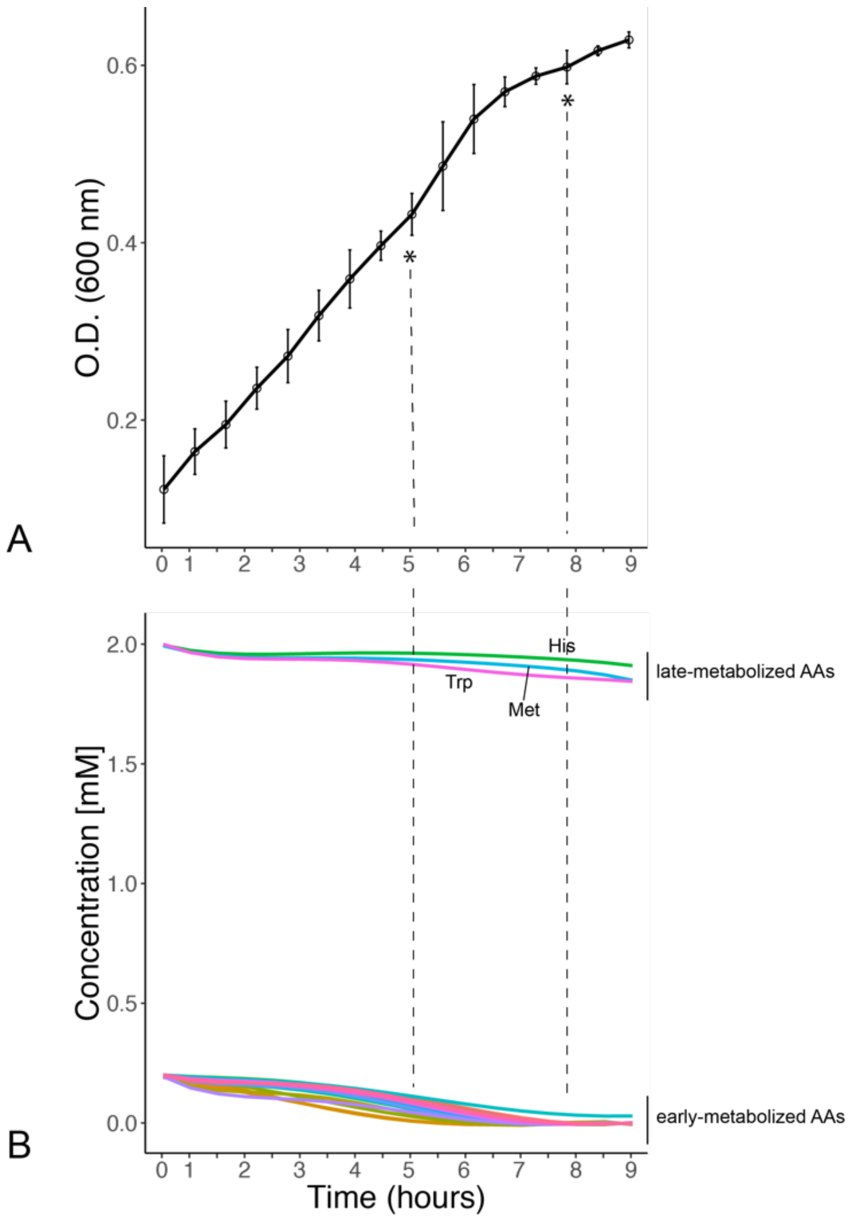

Supplementary Figure 9. Changing the concentration of late-metabolized amino acids. A) Growth curve of PhTAC125 grown in the 19 AA medium with Met, Trp and His provided at a concentration 10-times higher than the other amino acids (2mM in respect to 0.2 mM). Error bars represent SD of 2 different cell cultures in 2 independent experiments. Asterisks represent the growth lags. **B)** Degradation dynamics of the 19 amino acids and division between late- and early-metabolized compounds. Source data are provided as a Source Data file.

Supplementary Note 9. Amino acids consumption in *E. coli.*

In order to check whether the metabolic phenotype observed in PhTAC125 is specific of this microorganism or shared, for example, with the model system *E. coli*, we repeated the 19 AA experiment using the latter bacterium as our case-study. Overall (Supplementary Figure 10), we observed that the behaviour of *E. coli* in a nutritionally rich (but defined) medium differs from the one described for PhTAC125 in that: i) a large fraction of the amino acids

provided in the medium (9 out of 19) are scarcely or not used at all by *E. coli* as carbon/energy sources. These amino acids are the following: Val, Leu, Ile, Met, Asn, Lys, Tyr, His, Phe. ii) These amino acids include some of those that, on the contrary, are readily utilized by PhTAC125 (e.g. C2 amino acids Leu and Asn). iii) Other amino acids that are not used by PhTAC125 (e.g. Trp) are instead used by *E. coli* in its first growth stages. iv). No growth lags are observed during the growth of *E. coli* on the 19 AA medium, suggesting that no major switches in the uptake of nutrients occur.

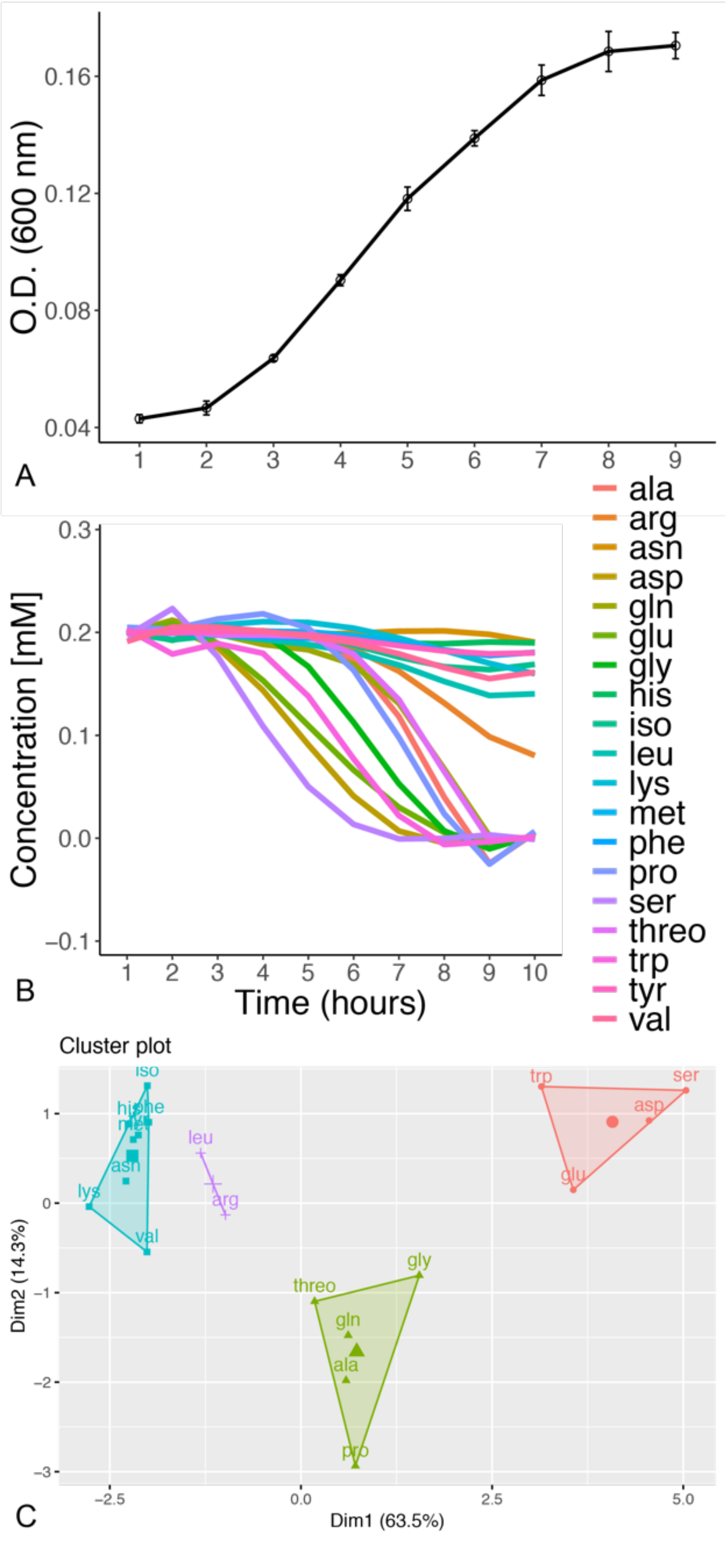

Supplementary Figure 10. The 19 AA experiment with *E. coli*. A) Growth curve of *E.coli* grown in the 19 AA medium. Error bars represent SD of 2 different cell cultures in 2 independent experiments. **B)** Degradation dynamics of the 19 amino acids, **C)** Clustering of time-resolved concentration values for the 19 amino acids analysed. Source data are provided as a Source Data file.

Similar to *PhTAC125*, a clustering algorithm identified 4 major clusters of catabolized amino acids, despite the elbow method criterion (Supplementary Figure 11) was ambiguous in the case of the *E. coli* experiment, partially supporting a separation between 2 amino acid clusters (catabolized vs. not catabolized amino acids).

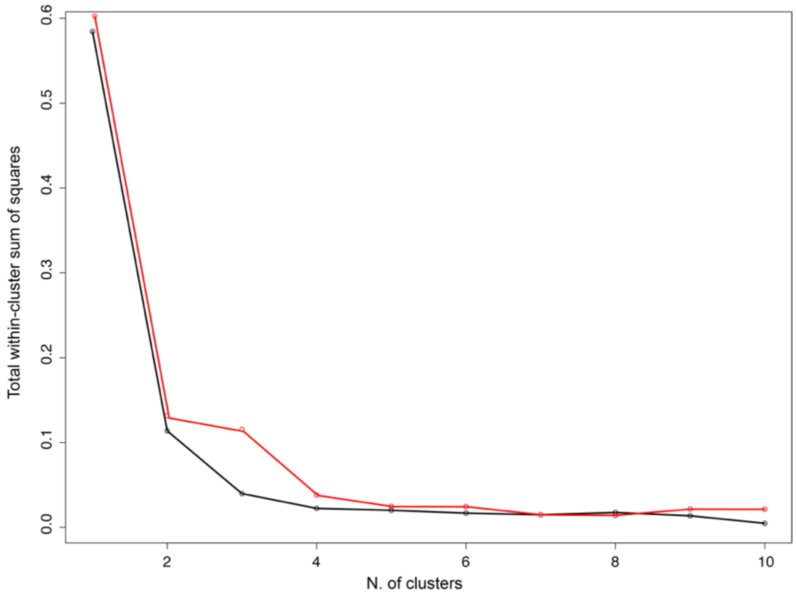

Supplementary Figure 11. Computing the number of actual clusters. A) Total within-cluster sum of squares (y-axis) and number of clusters selected for the k-means clustering of amino acids concentration patterns in the medium for PhTAC125 (black line) and *E. coli* (red line). 4 was chosen as the most significant number of clusters in both cases.

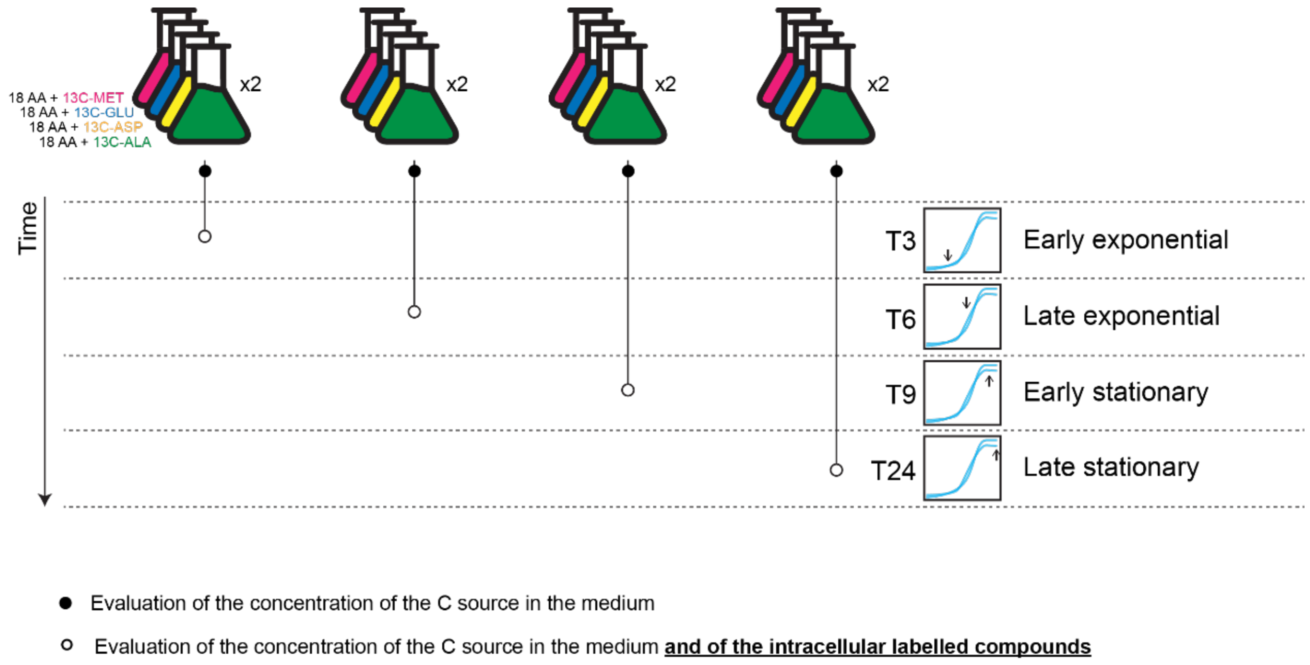

Supplementary Figure 12. The schematic representation of the ^{13}C labelled amino acids experiment. Four different experiments were performed using one different ^{13}C amino acid each time. For each experiment, two precultures as described in the main text were used, while the final growth experiments were performed in quadruplicate. Cell growth were monitored measuring the OD600 every hour. At four time point, early and late exponential growth (3 and 6 hours) and early and late stationary phase (8 hours and 30 minutes, 24 hours), one of the 4 replicates was analysed for both extracellular and intracellular metabolite content.

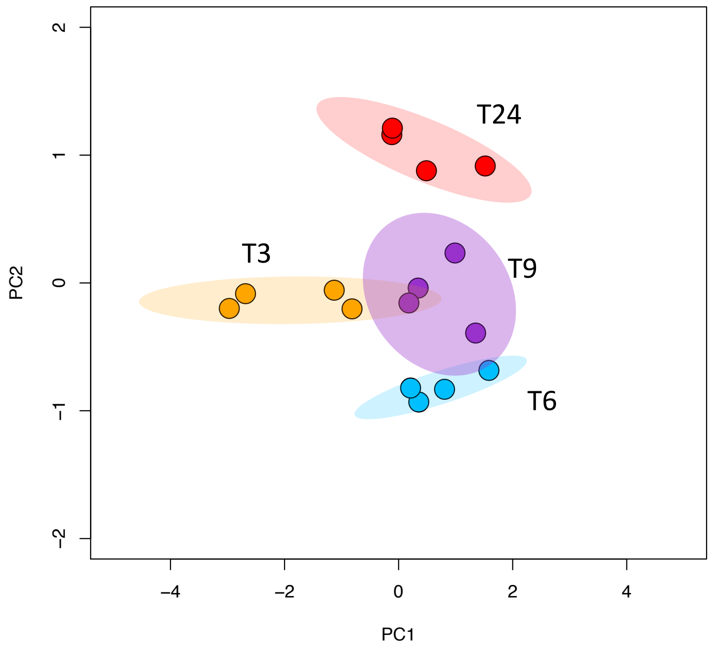

Supplementary Figure 13 Time points separation. PCA performed on the ^{1}H spectra of the experiment shown in Supplementary Figure 12. Labels on the different clusters refer to the sampling time points of the experiment (see Supplementary Figure 12).

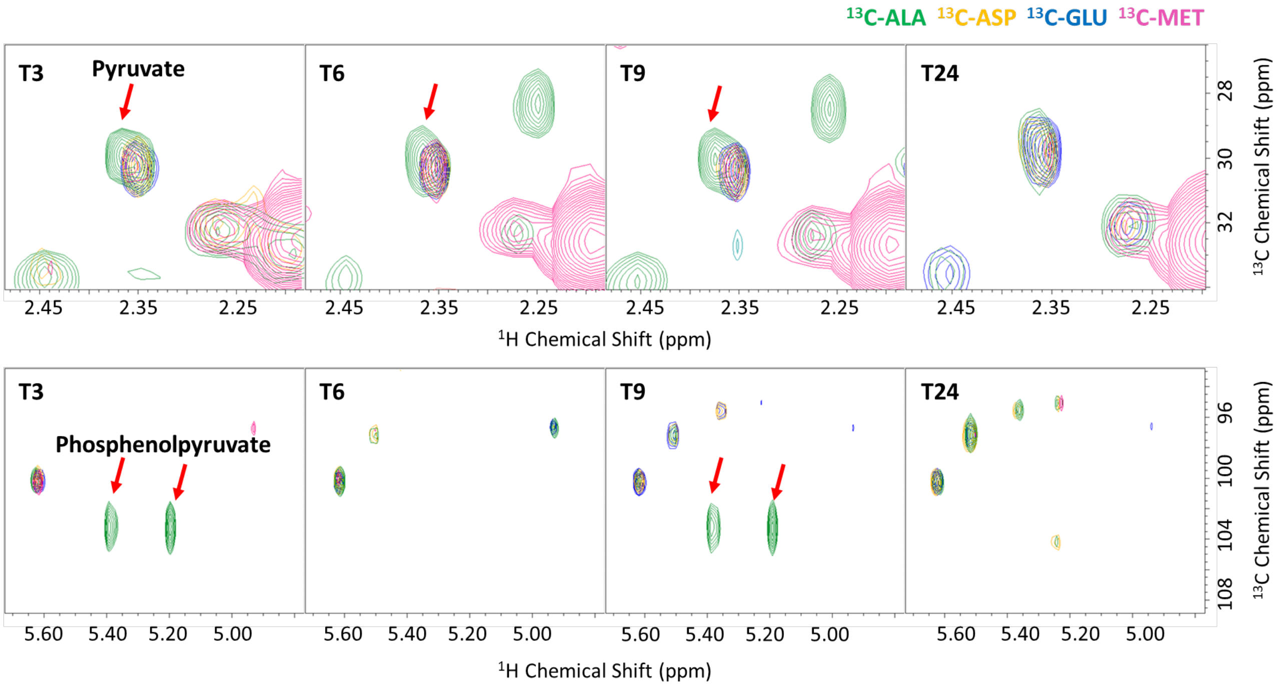

Supplementary Figure 14. ^{1}H-^{13}C HSQC signals of metabolites involved in glycolysis/gluconeogenesis, i.e. pyruvate and phosphoenolpyruvate. Signals from i) ^{13}C-Ala, green; ii) ^{13}C-Asp, yellow; iii) ^{13}C-Glu, green, iv) ^{13}C-Met, magenta.

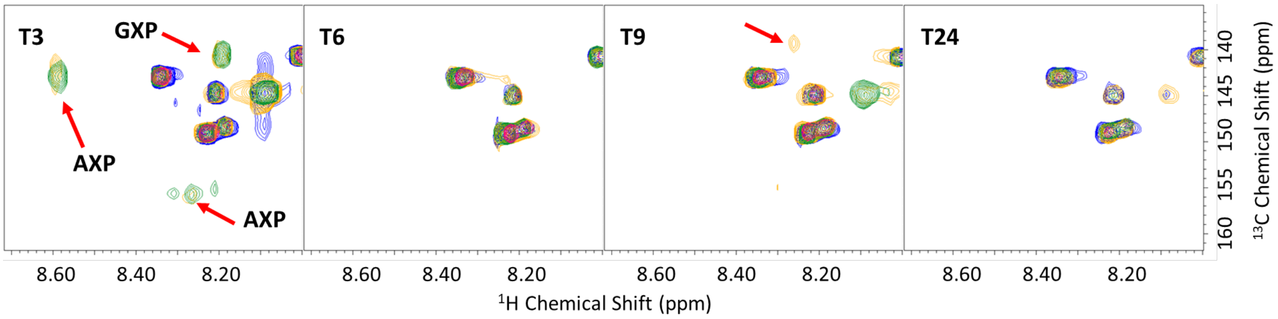

Supplementary Figure 15.^{1}H-^{13}C HSQC signals of nucleotides, i.e. AXP and GXP. Signals from i) ^{13}C-Ala, green; ii) ^{13}C-Asp, yellow; iii) ^{13}C-Glu, green, iv) ^{13}C-Met, magenta.

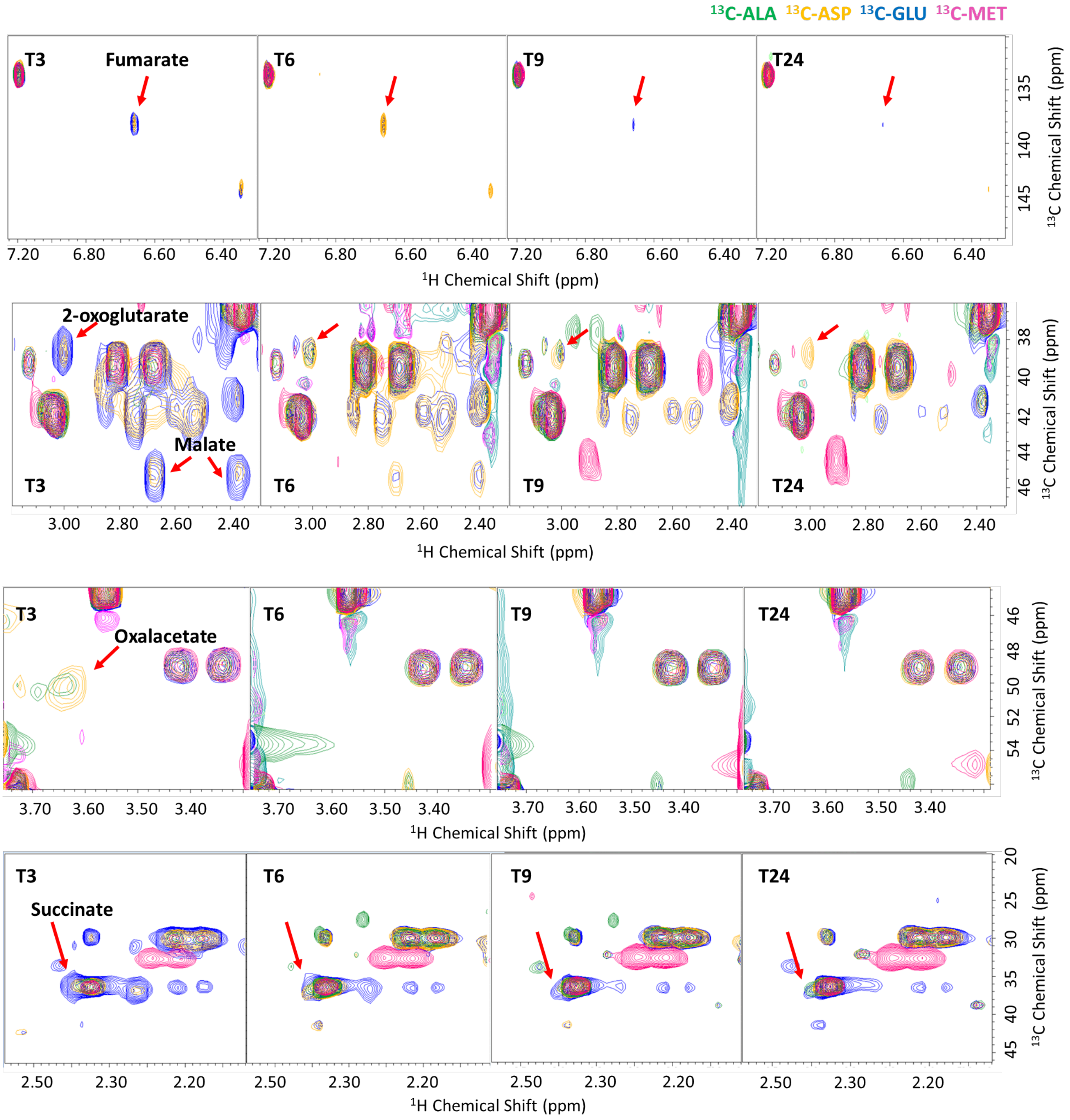

Supplementary Figure 16. ^{1}H-^{13}C HSQC signals of metabolites involved in TCA cycle, i.e. 2-oxoglutarate, malate, oxaloacetate and succinate. Signals from i) ^{13}C-Ala, green; ii) ^{13}C-Asp, yellow; iii) ^{13}C-Glu, green, iv) ^{13}C-Met, magenta.

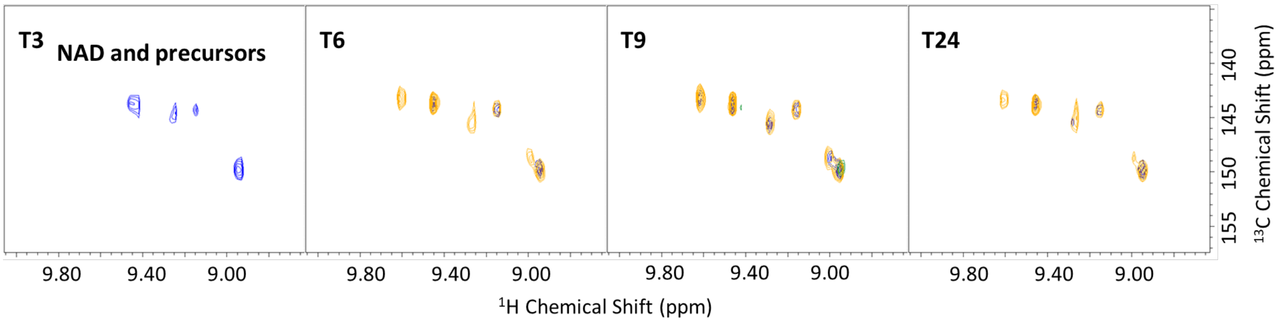

Supplementary Figure 17. ^{1}H-^{13}C HSQC signals of NAD and precursors. Signals from i) ^{13}C-Ala, green; ii) ^{13}C-Asp, yellow; iii) ^{13}C-Glu, green, iv) ^{13}C-Met, magenta.

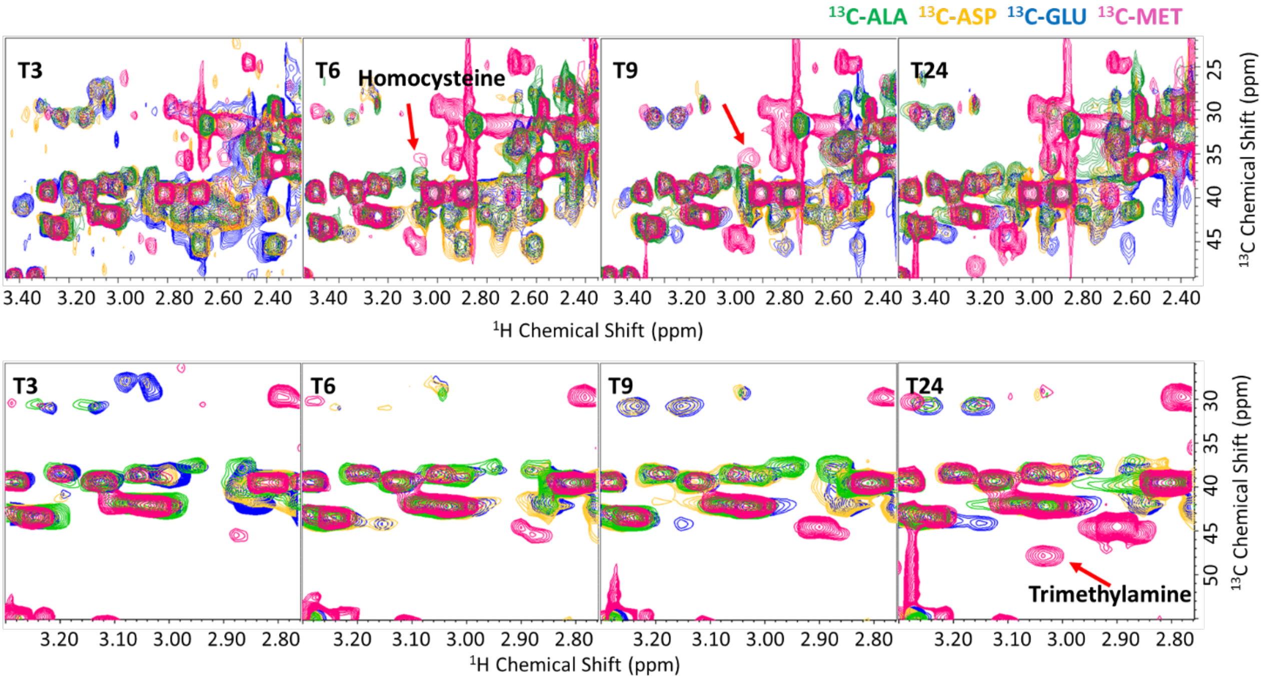

Supplementary Figure 18. ^{1}H-^{13}C HSQC signals of methionine related-metabolites, i.e. homocysteine and trimethylamine. Signals from i) ^{13}C-Ala, green; ii) ^{13}C-Asp, yellow; iii) ^{13}C-Glu, green, iv) ^{13}C-Met, magenta.

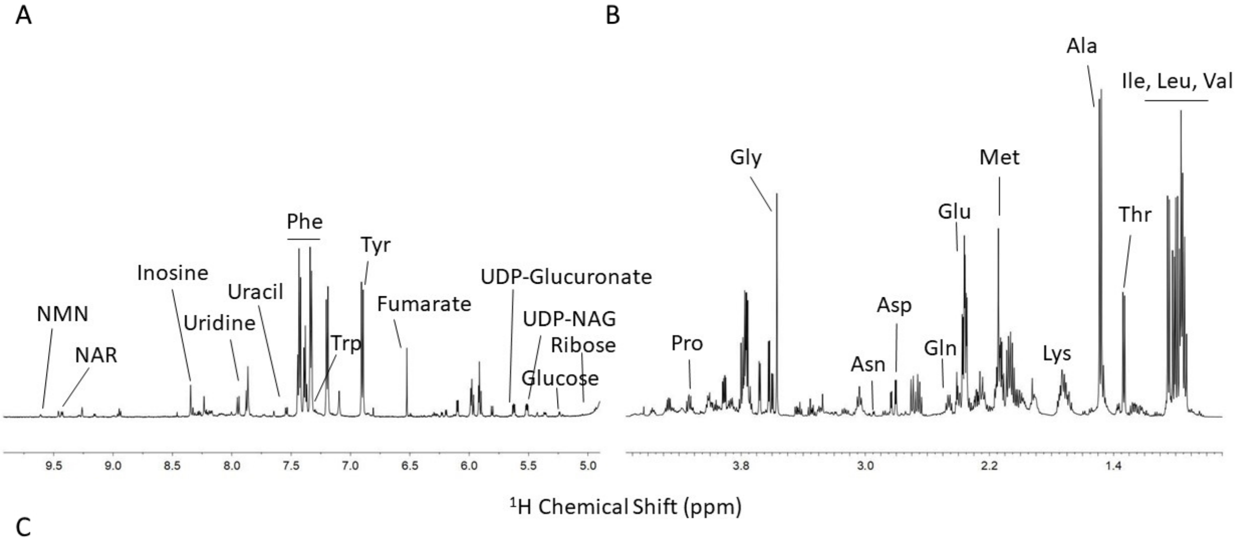

C

**Val**	0.98 (d)	1.03 (d)	2.26 (m)	3.60 (d)				
**Leu**	0.95(t)	1.70 (m)	3.72 (m)					
**Ile**	0.93 (t)	1.00 (d)	1.25 (m)	1.46 (m)	1.97 (m)	3.66 (d)		
**Thr**	1.32 (d)	3.58 (d)	4.24 (m)					
**Met**	2.16 (m)	2.63 (t)	3.85 (dd)					
**Ala**	1.47 (d)	3.77 (q)						
**Gln**	2.12 (m)	2.45 (m)	3.77 (t)					
**Glu**	2.04 (m)	2.12 (m)	2.34 (m)	3.75 (dd)				
**Asp**	2.67 (m)	2.83 (m)	3.87 (dd)					
**Asn**	2.84 (m)	2.94 (m)	4.00 (dd)					
**Pro**	2.00 (m)	2.08 (m)	2.34 (m)	3.32 (dt)	3.41 (m)	4.12 (dd)		
**Gly**	3.54 (s)							
**Tyr**	3.02 (dd)	3.17 (dd)	3.92 (dd)	6.88 (m)	7.17 (m)			
**Trp**	3.29 (dd)	3.47 (dd)	4.05 (dd)	7.19 (m)	7.27 (m)	7.31 (s)	7.53 (d)	7.72 (d)
**Phe**	3.11 (m)	3.27 (m)	3.98 (m)	7.37 (m)				
**Uridine**	3.80 (dd)	3.91 (dd)	4.12 (m)	4.22 (dd)	4.34 (dd)	5.89 (d)	5.90 (d)	7.86 (d)
**Inosine**	3.83 (dd)	3.90 (dd)	4.26 (dd)	4.42 (dd)	4.80 (s)	6.05 (d)	8.19 (s)	8.30 (s)
**Uracil**	5.79 (d)	7.52 (d)						
**UDP-Glucuronate**	3.51 (t)	3.58 (m)	3.77 (t)	4.13 (d)	4.19 (m)	4.27 (m)	4.34 (m)	5.61 (dd)
	5.97 (m)	7.92 (d)						
**UDP-NAG**	2.07 (s)	3.54 (t)	3.80 (m)	3.91 (m)	3.98 (m)	4.17 (m)	4.23 (m)	4.27 (m)
	4.35 (m)	5.50 (dd)	5.96 (m)	7.93 (t)	8.32 (d)			
**Formate**	8.45 (s)							
**Fumarate**	6.51 (s)							
**#Glucose**	3.23 (dd)	3.40 (m)	3.46 (m)	3.52 (dd)	3.73 (m)	3.82 (m)	3.89 (dd)	4.63 (d)
	5.22 (d)							
**#Ribose**	3.51 (dd)	3.60 (dd)	3.67 (dd)	3.86 (m)	3.97 (m)	4.09 (m)	4.20 (dd)	4.92 (d)
	5.25 (d)	5.37 (d)						
***NAR**	6.23 (d)	8.20 (t)	8.95 (d)	9.16 (d)	9.47 (s)			
***NMN**	6.20 (d)	8.32 (t)	9.00 (d)	9.34 (d)	9.60 (s)			

Supplementary Figure 19. Metabolomic summary data for the main assignments. A) Upfield (1.00–4.50 ppm) and B) downfield (5.00–10.00 ppm) region of the ^{1}H NMR CPMG spectrum of cell lysate. C) 1H NMR resonance assignment for the 19 amino acid' signals. s= singlet; d= doublet; t=triplet; dd= doublet of doublets; dt= doublet of triplets; m=multiplet.
Assignment confirmed by spiking
*Assignment confirmed by literature data [12]

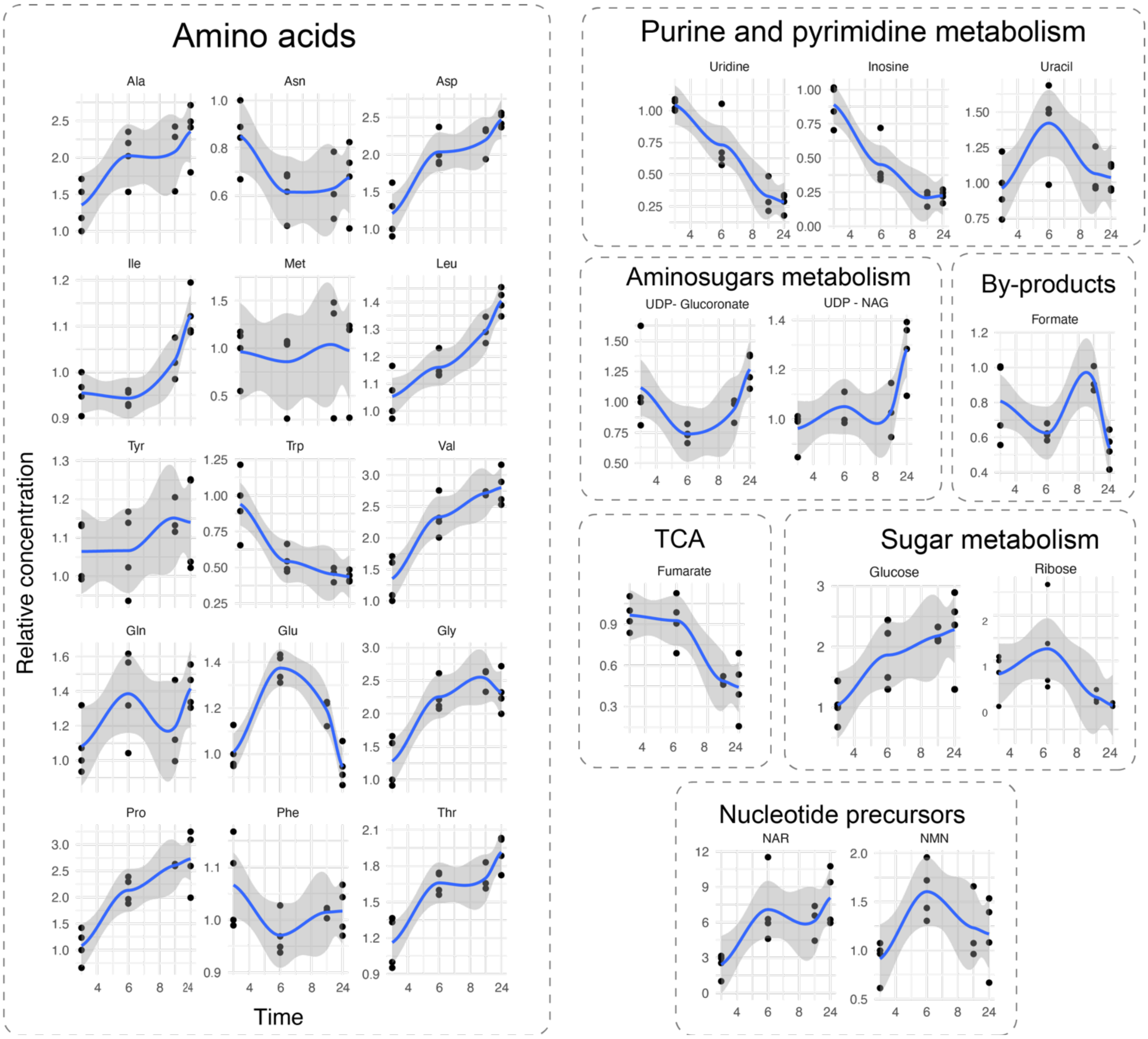

Supplementary Figure 20. Metabolites concentration in time. Relative concentration changes for 26 intracellular metabolites. For each metabolite, the relative concentration was obtained dividing its concentration at each time point by the concentration of the same metabolite at T3. Source data are provided as a Source Data file. Grey shaded area includes the 95% confidence of the linear regression (coloured) line over the concentrations of the compounds in 4 cultures in 2 independent experiments.

Supplementary Table 7. Parameters used in this work for the MMM model

Parameter	Description	Value	Units
β_1	max rate constant of *P* on S_1	0.0919	$g/g_{CDW}*h$
β_2	max rate constant of *P* on S_2	0.0478	$g/g_{CDW}*h$
β_3	max rate constant of *P* on S_3	0.3101	$g/g_{CDW}*h$
β_4	max rate constant of *P* on S_4	0.0277	$g/g_{CDW}*h$
k_1	Michaelis-Menten constant for S_1	0.0497	g/l
k_2	Michaelis-Menten constant for S_2	0.0268	g/l

k_3	Michaelis-Menten constant for S_3	2.9170	g/l
k_4	Michaelis-Menten constant for S_4	3.3195	g/l
d	Death rate	0.001	h^{-1}

Supplementary Table 8. Parameters used in this work for the cybernetic model

Parameter	Description	Value	Units
V_{max1}	max rate constant of *P* on S_1	0.20341	$g/g_{CDW}*h$
V_{max2}	max rate constant of *P* on S_2	2.3651	$g/g_{CDW}*h$
V_{max3}	max rate constant of *P* on S_3	160.45	$g/g_{CDW}*h$
V_{max4}	max rate constant of *P* on S_4	0.35559	$g/g_{CDW}*h$
K_{s1}	Michaelis-Menten constant for S_1	182.06	g/l
K_{s2}	Michaelis-Menten constant for S_2	5.0622	g/l
K_{s3}	Michaelis-Menten constant for S_3	4484.6	g/l
K_{s4}	Michaelis-Menten constant for S_4	39.41	g/l
V_{e1}	max rate constant of e_1 synthesis	1.8702	$g/g_{CDW}*h$
V_{e2}	max rate constant of e_2 synthesis	1.0252e-05	$g/g_{CDW}*h$
V_{e3}	max rate constant of e_3 synthesis	0.020896	$g/g_{CDW}*h$
V_{e4}	max rate constant of e_4 synthesis	0.014356	$g/g_{CDW}*h$
K_{e1}	Michaelis-Menten constant for e_1	0.0034958	g/l
K_{e2}	Michaelis-Menten constant for e_2	2.0675e-08	g/l
K_{e3}	Michaelis-Menten constant for e_3	8.4553e-13	g/l
K_{e4}	Michaelis-Menten constant for e_4	2.4577e-08	g/l
d	Death rate	0.001	h^{-1}
α	Enzyme dilution rate	0.0098085	h^{-1}
λ	P fraction increase due to consumption of S	1	-
β	Basic enzyme synthesis rate	0.001	h^{-1}

Supplementary Note 10. Main players in amino acids assimilation.

We selected one amino acid for each of the four groups (Glu, Asp, Phe and Met) and analysed the expression (delta-CT values) of those genes that i) were involved in the their possible first assimilatory steps (Figure 2D) and ii) resulted to be differentially expressed in, at least, one contrast of the differential transcriptomic analysis. Data obtained revealed interesting insights on the regulation of amino acid metabolism during nutrients switching. Glutamine synthetase (*glnA*), for example, shows an increase in its expression until the first lag point (4 hours); afterwards, its expression decreases (Supplementary Figure 21). GlnA uses Glu as substrate, thus this expression trend is consistent with the concentration of Glu dropping close to zero after four hours of growth. The expression increase in the first stage of the growth can be explained by the ongoing exhaustion of Gln in the medium and the necessity to synthesize it from Glu. Further, *asnB* encodes an asparagine synthetase B and is involved in the formation of Asn from Asp. The expression of this gene dramatically increases following the second short lag phase reported (i.e. after 6 hours of growth) (Supplementary Figure 21). This is consistent with the exhaustion of Asn in the medium at that time point and with the necessity to synthesize this amino acid, i.e. adding an amino group to Asp that, in turn, can be synthesized from TCA intermediates (e.g. oxaloacetate). *metK* (Met degradation) displays a trend that is characterized by an initial increase of its expression, followed by a down regulation in correspondence of the first growth lag phase, and a final expression increase consistent with the necessity to actively exploit these carbon and energy sources when most of the others are depleted. Finally, we confirmed the complementary expression pattern of *metK* and *mdeA*, responsible for the entrance of Met

degradation intermediates into sulfur metabolism and for the conversion of Met to S-adenosyl-methionine (SAM), respectively (Supplementary Figure 21).

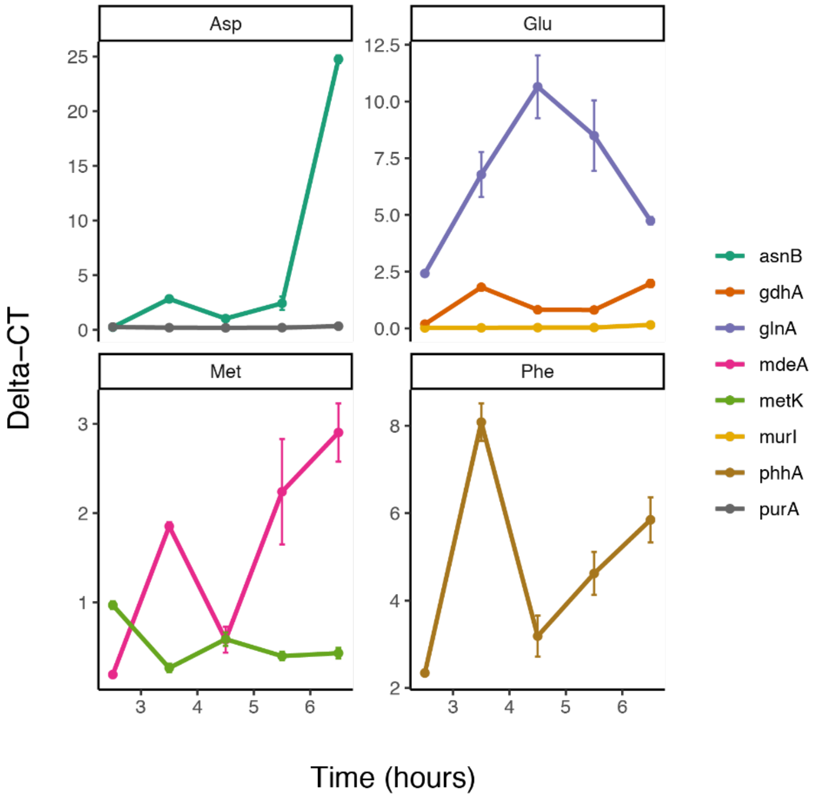

Supplementary Figure 21. Real time PCR data Expression (delta-CT) values for the genes involved in the first assimilatory steps of 4 selected amino acids. Source data are provided as a Source Data file. Error bars represent SD of 2 different cell cultures in 2 independent experiments.

Supplementary Table 9. Features of the RT primers used in this work

Primer	Sequence	Tm (°C)	Lenght (bp)	GC%	Amplicon size (bp)	Source
metK_TAC_for	5'-CGTACTAGCCCAGAAGAGCA-3'	58.90	20	55.00	160	This work
metK_TAC_rev	5'-GTAACCAGTTAAGCTCGCCG-3'	59.00	20	55.00		This work
glnA_TAC_for	5'-TCAATTGCTGGCTGGAAAGG-3'	58.74	20	50.00	151	This work
glnA_TAC_rev	5'-CGCGCTCGTAACCTTGTAAT-3'	58.73	20	50.00		This work
purA_TAC_for	5'-GCGCACAAGGTACGTTACTT-3'	58.86	20	50.00	164	This work
purA_TAC_rev	5'-GCCTGAACCAACACGTGTAG-3'	59.13	20	55.00		This work
murI_TAC_for	5'-ATCAAGTCCCTATACGCGCA-3'	58.96	20	50.00	150	This work
murI_TAC_rev	5'-CGATCGAGTTCTTGGTGCAG-3'	59.01	20	55.00		This work
gdhA_TAC_for	5'-GCTCTACCTTGGGCCCATAT-3'	58.93	20	55.00	151	This work
gdhA_TAC_rev	5'-GGATCAAAGTTAGCACCGCC-3'	59.27	20	55.00		This work
phhA_TAC_for	5'-AATTTCCGGTGGCGACTTTT-3'	58.68	20	45.00	188	This work
phhA_TAC_rev	5'-CATGCGCGCTAGGTATACAC-3'	58.94	20	55.00		This work

mdeA_TAC_for	5'-CGGTATCGGCCTCAGTACTT-3'	58.97	20	55.00	160	This work
mdeA_TAC_rev	5'-GCGCGTAATTCATCCTCGTT-3'	59.07	20	50.00		This work
asnB_TAC_for	5'-TGATCCCGTACAGTTGCGTA-3'	58.82	20	50.00	157	This work
asnB_TAC_rev	5'-ATATAAAGGCTGTGCACCGC-3'	58.69	20	50.00		This work
sdaA_TAC_for	5'-TGCTCACCACTACCACGAAT-3'	59.03	20	50.00	155	This work
sdaA_TAC_rev	5'-GAAAATGCCGCCGAAATTGG-3'	59.00	20	50.00		This work
glyA_TAC_for	5'-AATAGCTTCGTCGCCACATG-3'	58.71	20	50.00	151	This work
glyA_TAC_rev	5'-TGTAGATATGGCGCACGTTG-3'	58.43	20	50.00		This work
rplM_TAC_for	5'-TGATAAACTTCAAGCTGCAAAGC-3'	58.45	23	39.13	152	This work
rplM_TAC_rev	5'-GAACCTGAGGCTGTTGTGC-3'	59.05	19	57.89		This work
dnaA_TAC_for	5'-GCTAACAAAGAGCGCTCACA-3'	58.85	20	50.00	184	This work
dnaA_TAC_rev	5'-GTGTTTCAAGCTCAGGAGGC-3'	59.12	20	55.00		This work

Supplementary References

1. Williams M. D., Ouyang T. X., Flickinger M. C. Starvation-induced expression of SspA and SspB: the effects of a null mutation in sspA on Escherichia coli protein synthesis and survival during growth and prolonged starvation. *Mol Microbiol* **11**, 1029-1043 (1994).

2. Mannan A. A., Toya Y., Shimizu K., McFadden J., Kierzek A. M., Rocco A. Integrating Kinetic Model of E. coli with Genome Scale Metabolic Fluxes Overcomes Its Open System Problem and Reveals Bistability in Central Metabolism. *PLoS One* **10**, e0139507 (2015).

3. Gohler A. K*., et al.* More than just a metabolic regulator--elucidation and validation of new targets of PdhR in Escherichia coli. *BMC systems biology* **5**, 197 (2011).

4. Galperin M. Y., Nikolskaya A. N., Koonin E. V. Novel domains of the prokaryotic two-component signal transduction systems. *FEMS microbiology letters* **203**, 11-21 (2001).

5. Aldridge P., Paul R., Goymer P., Rainey P., Jenal U. Role of the GGDEF regulator PleD in polar development of Caulobacter crescentus. *Mol Microbiol* **47**, 1695-1708 (2003).

6. Huang B., Whitchurch C. B., Mattick J. S. FimX, a multidomain protein connecting environmental signals to twitching motility in Pseudomonas aeruginosa. *J Bacteriol* **185**, 7068-7076 (2003).

7. Wilde A., Fiedler B., Borner T. The cyanobacterial phytochrome Cph2 inhibits phototaxis towards blue light. *Mol Microbiol* **44**, 981-988 (2002).

8. Boles B. R., McCarter L. L. Vibrio parahaemolyticus scrABC, a novel operon affecting swarming and capsular polysaccharide regulation. *J Bacteriol* **184**, 5946-5954 (2002).

9. Papa R., Rippa V., Duilio A. Identification of the transcription factor responsible for L-malate-dependent regulation in the marine Antarctic bacterium Pseudoalteromonas haloplanktis TAC125. *FEMS microbiology letters* **295**, 177-186 (2009).

10. Wilmes B., Hartung A., Lalk M., Liebeke M., Schweder T., Neubauer P. Fed-batch process for the psychrotolerant marine bacterium Pseudoalteromonas haloplanktis. *Microb Cell Fact* **9**, 72 (2010).

11. Fondi M., Bosi E., Presta L., Natoli D., Fani R. Modelling microbial metabolic rewiring during growth in a complex medium. *BMC Genomics* **17**, 970 (2016).

12. Shabalin K., *et al.* NAD Metabolome Analysis in Human Cells Using (1)H NMR Spectroscopy. *Int J Mol Sci* **19**, (2018).
