# Supplementary material for: Diauxie and co-utilization of carbon sources can coexist during bacterial growth in nutritionally complex environments
Source: Nat Commun. 2020 Jun 19;11:3135. doi: 10.1038/s41467-020-16872-8 (PMC7305145; doi:10.1038/s41467-020-16872-8)
Supplement: Supplementary file 3 — Description of Additional Supplementary Files [file 41467_2020_16872_MOESM3_ESM.pdf]

Description of Additional Supplementary Files

File Name: Supplementary Data 1
Description: *E. coli* global regulators known to be involved in metabolism control in E. coli and their corresponding PhTAC125 ortholog.

File Name: Supplementary Data 2
Description: List of differentially expressed PhTAC125 TFs identified. All differentially expressed TS were identified following the T1-T3 transition, except * that was identified following the T3-T5 one. Adjusted p-values were calculated using Deseq2 default approach, i.e. correcting for multiple testing using the Benjamini and Hochberg method.

File Name: Supplementary Data 3
Description: List of differentially expressed *Ph*TAC125 TCSRs identified. All differentially expressed TS were identified following the T1-T3 transition, except * that was also identified following the T3-T5 one (log_2FC and p-value are provided in the same line). Adjusted p-values were calculated using Deseq2 default approach, i.e. correcting for multiple testing using the Benjamini and Hochberg method.

File Name: Supplementary Data 4
Description: List of genes responsible for the first assimilation step of amino acids in *Ph*TC125.
