# Supplementary material for: Diauxie and co-utilization of carbon sources can coexist during bacterial growth in nutritionally complex environments
Source: Nat Commun. 2020 Jun 19;11:3135. doi: 10.1038/s41467-020-16872-8 (PMC7305145; doi:10.1038/s41467-020-16872-8)
Supplement: Supplementary file 8 — Reporting Summary [file 41467_2020_16872_MOESM8_ESM.pdf]

nature research

Corresponding author(s): Marco Fondi
Last updated by author(s): 2020/05/12

Reporting Summary

Nature Research wishes to improve the reproducibility of the work that we publish. This form provides structure for consistency and transparency in reporting. For further information on Nature Research policies, see our Editorial Policies and the Editorial Policy Checklist.

Statistics

For all statistical analyses, confirm that the following items are present in the figure legend, table legend, main text, or Methods section.

n/a	Confirmed	
☐	☒	The exact sample size (n) for each experimental group/condition, given as a discrete number and unit of measurement
☐	☒	A statement on whether measurements were taken from distinct samples or whether the same sample was measured repeatedly
☐	☒	The statistical test(s) used AND whether they are one- or two-sided<br>*Only common tests should be described solely by name; describe more complex techniques in the Methods section.*
☒	☐	A description of all covariates tested
☒	☐	A description of any assumptions or corrections, such as tests of normality and adjustment for multiple comparisons
☐	☒	A full description of the statistical parameters including central tendency (e.g. means) or other basic estimates (e.g. regression coefficient) AND variation (e.g. standard deviation) or associated estimates of uncertainty (e.g. confidence intervals)
☐	☒	For null hypothesis testing, the test statistic (e.g. F, t, r) with confidence intervals, effect sizes, degrees of freedom and P value noted<br>*Give P values as exact values whenever suitable.*
☒	☐	For Bayesian analysis, information on the choice of priors and Markov chain Monte Carlo settings
☒	☐	For hierarchical and complex designs, identification of the appropriate level for tests and full reporting of outcomes
☐	☒	Estimates of effect sizes (e.g. Cohen's d, Pearson's r), indicating how they were calculated

Our web collection on statistics for biologists contains articles on many of the points above.

Software and code

Policy information about availability of computer code

Data collection	Software to generate and perform simulations was developed and is available on Sourceforge as specified in the manuscript in the "code availability" section.
Data analysis	The following tools were used during data analysis: Bowtie 2 (v2.2.3), blastall v. 2.2.26, MAST(version 5.0.5), MEME suite v. 5.1.1, Primer3 4.1.0, MATLAB 2019a, DeSeq2 Release 3.11,

For manuscripts utilizing custom algorithms or software that are central to the research but not yet described in published literature, software must be made available to editors and reviewers. We strongly encourage code deposition in a community repository (e.g. GitHub). See the Nature Research guidelines for submitting code & software for further information.

Data

Policy information about availability of data

All manuscripts must include a data availability statement. This statement should provide the following information, where applicable:

- Accession codes, unique identifiers, or web links for publicly available datasets
- A list of figures that have associated raw data
- A description of any restrictions on data availability

RNAseq data that support the findings of this study have been deposited in NCBI SRA archive with the accession codes SAMN12207305 to SAMN12207324. Metabolomics data have been deposited at MetaboLigths (https://www.ebi.ac.uk/metabolights/) under code unique identifier MTBLS1699 (www.ebi.ac.uk/metabolights/MTBLS1699).

Databases used in this work: COG (https://www.ncbi.nlm.nih.gov/COG/), KEGG (https://www.genome.jp/kegg/) , RegPrecise (http://regprecise.sbpdiscovery.org:8080/WebRegPrecise/).

The authors declare that the other data supporting the findings of this study are available within the paper. No restrictions apply to data availability. The source data underlying Figures 1a-c, 2a-d, 3b-e, 4a, c and d, 5a, b, S6a, S8a-d, S9a, b, 10a, b, S20 and S21are provided as a Source Data files.

Field-specific reporting

Please select the one below that is the best fit for your research. If you are not sure, read the appropriate sections before making your selection.

[x] Life sciences   [ ] Behavioural & social sciences   [ ] Ecological, evolutionary & environmental sciences

For a reference copy of the document with all sections, see nature.com/documents/nr-reporting-summary-flat.pdf

Life sciences study design

All studies must disclose on these points even when the disclosure is negative.

Sample size	No sample-size calculation. This is not an empirical study in which the goal is to make inferences about a population from a sample. The entire populations studied in this work were pooled and analyzed as a whole.
Data exclusions	No data were excluded from the analysis
Replication	For each experiment (at least) two independent biological and techincal replicates were performed
Randomization	The manuscript deals with microbial growth experiments where randomizations are not a valid techique (see also reply to point 1)
Blinding	The manuscript deals with microbial growth experiments and computational simulations where blinding cannot be applied (see also reply to point 1)

Reporting for specific materials, systems and methods

We require information from authors about some types of materials, experimental systems and methods used in many studies. Here, indicate whether each material, system or method listed is relevant to your study. If you are not sure if a list item applies to your research, read the appropriate section before selecting a response.

Materials & experimental systems

n/a	Involved in the study	
[x]	[ ]	Antibodies
[x]	[ ]	Eukaryotic cell lines
[x]	[ ]	Palaeontology and archaeology
[x]	[ ]	Animals and other organisms
[x]	[ ]	Human research participants
[x]	[ ]	Clinical data
[x]	[ ]	Dual use research of concern

Methods

n/a	Involved in the study	
[x]	[ ]	ChIP-seq
[x]	[ ]	Flow cytometry
[x]	[ ]	MRI-based neuroimaging
